# A robot-aided visuomotor wrist training induces gains in proprioceptive and movement accuracy in the contralateral wrist

**DOI:** 10.1038/s41598-021-84767-9

**Published:** 2021-03-05

**Authors:** Yizhao Wang, Huiying Zhu, Naveen Elangovan, Leonardo Cappello, Giulio Sandini, Lorenzo Masia, Jürgen Konczak

**Affiliations:** 1 Department of Rehabilitation Medicine, Tianjin Huanhu Hospital, Tianjin, China; 2 Human Sensorimotor Control Laboratory, School of Kinesiology, University of Minnesota, Minneapolis, USA; 3 College of Exercise and Health Sciences, Tianjin University of Sport, Tianjin, China; 4 The BioRobotics Institute, Scuola Superiore Sant’Anna, Pisa, Italy; 5 Department of Excellence in Robotics and AI, Pisa, Italy; 6 Department of Robotics, Brain and Cognitive Sciences, Istituto Italiano di Tecnologia, Genova, Italy; 7 Institut für Technische Informatik, Universität Heidelberg, Heidelberg, Germany

**Keywords:** Sensorimotor processing, Somatosensory system

## Abstract

Proprioceptive training is a neurorehabilitation approach known to improve proprioceptive acuity and motor performance of a joint/limb system. Here, we examined if such learning transfers to the contralateral joints. Using a robotic exoskeleton, 15 healthy, right-handed adults (18–35 years) trained a visuomotor task that required making increasingly small wrist movements challenging proprioceptive function. Wrist position sense just-noticeable-difference thresholds (JND) and spatial movement accuracy error (MAE) in a wrist-pointing task that was not trained were assessed before and immediately as well as 24 h after training. The main results are: first, training reduced JND thresholds (− 27%) and MAE (− 33%) in the trained right wrist. Sensory and motor gains were observable 24 h after training. Second, in the untrained left wrist, mean JND significantly decreased (− 32%) at posttest. However, at retention the effect was no longer significant. Third, motor error at the untrained wrist declined slowly. Gains were not significant at posttest, but MAE was significantly reduced (− 27%) at retention. This study provides first evidence that proprioceptive-focused visuomotor training can induce proprioceptive and motor gains not only in the trained joint but also in the contralateral, homologous joint. We discuss the possible neurophysiological mechanism behind such sensorimotor transfer and its implications for neurorehabilitation.

## Introduction

Proprioceptive signals from peripheral mechanoreceptors are crucial for motor control and form the basis for movement coordination, postural control, and body awareness. Proprioceptive deficits can severely impair motor control. They are common in neurological conditions such as stroke^1^ and Parkinson’s disease^2^. Somatosensory-based interventions can improve proprioceptive function and thereby, lead to improvements in motor function^3^.

In this context, proprioceptive training is an intervention aimed at improving sensorimotor function by focusing on proprioceptive afferent signals over feedback from other sensory modalities such as vision and audition^3^. Previous work from our group demonstrated that a short proprioceptive-focused intervention of less than an hour enhances wrist proprioceptive acuity in healthy as well as clinical population such as Parkinson’s disease and stroke survivors^4–7^. Importantly, these studies also showed that such somatosensory-based training also improves the movement accuracy in an untrained motor task involving the same joint. That is, it induces a sensory as well as a desired motor effect. Moreover, visuomotor training has been shown to enhance proprioceptive function^8^. The task employed in our previous research differs from classic visuomotor training as it requires to make increasingly small, rapid online movement error corrections that have been shown to challenge the proprioceptive system^9^. For the sake of brevity, we here refer to such training as *visuomotor training* and acknowledge that the underlying processes of visuo-proprioceptive integration contribute to motor learning in this task.

Short- and long-term neuroplastic changes in cortical areas have been reported as the result of proprioceptive-focused sensorimotor learning. For example, following sensorimotor learning, the latency of somatosensory evoked potentials (SEPs) reduces in somatosensory cortical areas of the brain and these changes are reliably correlated with the magnitude of motor learning: participants who learned more showed greater changes in SEP magnitude^10^. Moreover, functional brain imaging revealed a functional reorganization within the primary sensorimotor cortex and supplementary motor area following four weeks of proprioceptive training^11^.

Numerous studies established the notion of interlimb transfer of motor learning^12–15^. That is to say, the untrained contralateral limb exhibits signs of motor learning without practice. Specifically, studies on force and visuomotor adaptations demonstrated that adaptive learning can lead to observable changes in the movement kinematics and kinetics of the untrained limb^16,17^. While there is evidence for transfer of motor learning to the untrained side, a transfer of somatosensory learning has not been evaluated systematically. It is unknown whether the improvements in proprioception and motor function due to visuomotor training transfers to a contralateral and homologous joint. If such contralateral transfer of proprioceptive learning is observed, such finding can impact neurological rehabilitation. For instance, it is very challenging to deliver physical therapy in the affected joints on stroke survivors with unilateral proprioceptive and motor dysfunction. In such cases, an interlimb transfer of somatosensory learning will allow training to be delivered on the patient’s less or unaffected side to facilitate improvements in proprioceptive and motor function on the affected side.

To address these knowledge gaps, we employed a brief, single session right wrist visuomotor training and evaluated wrist sensory and motor function bilaterally for three consecutive days. Healthy adult participants used a robotic wrist exoskeleton to practice a visuomotor task that required making increasingly precise, small amplitude wrist movements challenging proprioception. Using the same robot, we determined the *Just-Noticeable Difference* (JND) position sense threshold as a marker of proprioceptive acuity (i.e. the ability to discriminate between two joint positions). For this test, vision was occluded and the robot moved the wrist/hand passively. In addition, participants performed a goal-directed wrist-pointing task before and after training. We assessed the spatial *Movement Accuracy Error* (MAE) for this untrained motor task to determine if learning had extended to the untrained motor domain. Thus, the main aim of the study was to determine, if sensory and motor learning that had occurred in the trained right wrist transferred to the untrained left wrist, and whether such learning transfer was still retained after 24 h.

## Results

### Evidence of task-specific motor learning during visuomotor training

**Figure 1:**
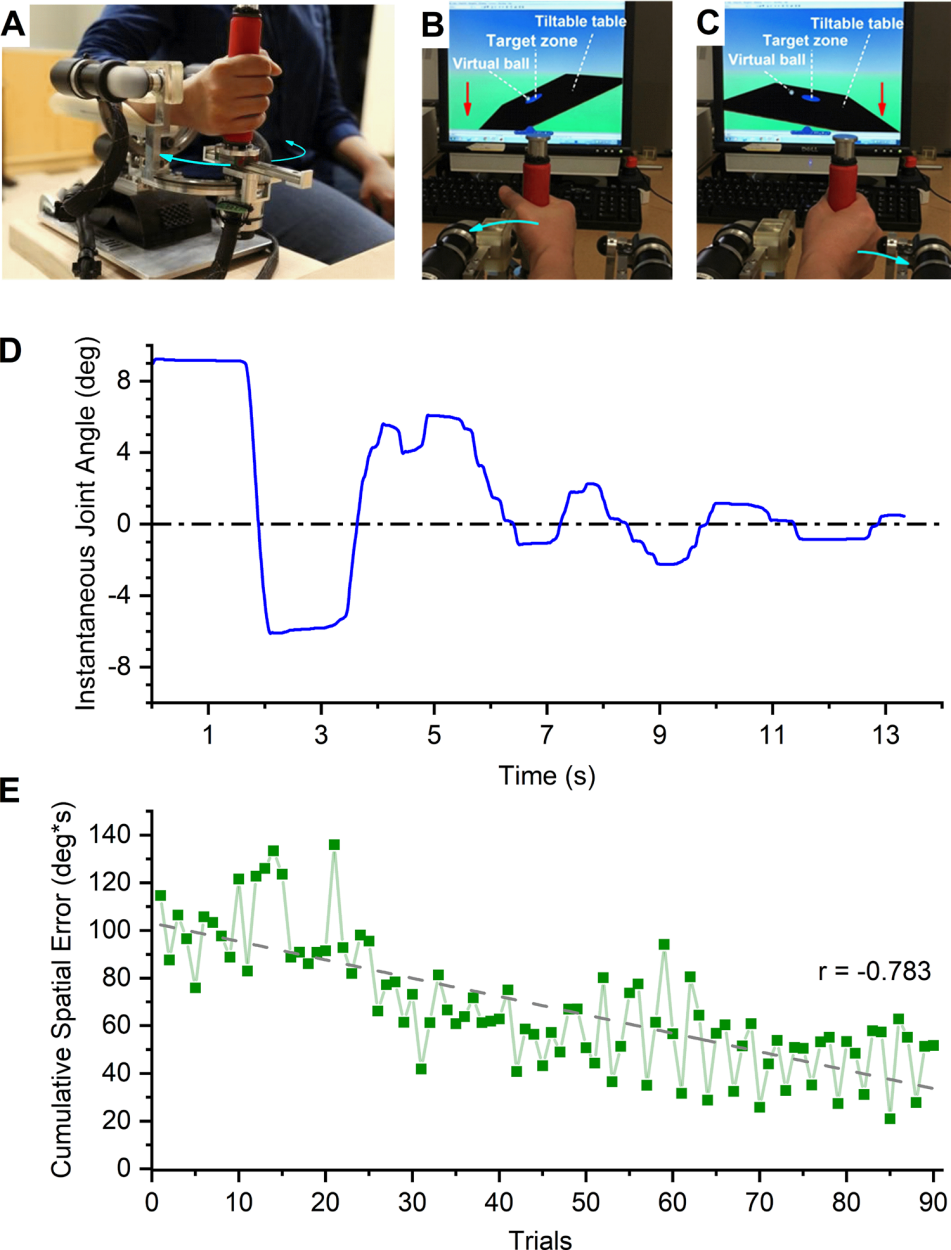
Wrist robot and visuomotor training. (**A)** Antero-lateral view of the wrist robot with a participant's forearm positioned in the support splint, hand holding the device handle. (**B)** Visual display of the task. Goal is to bring the ball rolling on the virtual table into the target zone. Wrist flexion tilts the virtual table to the left. (**C)** Wrist extension tilts the virtual table to the left. (**D)** Instantaneous wrist flexion/extension joint angle changes during a single training trial from one of the participants. (**E)** Learning effect on movement trajectory formation as measured by the Cumulative Spatial Error (CSE). Each data point represents the mean CSE of all participants for a particular trial. Note the decline of CSE over successive trials. Dashed line indicates the fit of a linear regression model. *r* coefficient of correlation.

To obtain a marker of motor learning due to the robot-aided visuomotor training we determined the Cumulative spatial error (CSE) of the wrist joint trajectory for each trial (see Fig. 1A–C for the setup and “Methods” for a detailed description of the training procedure). Most participants (13/15) finished the 90 training trials in 45 min. After hitting the target at each amplitude at least once, the level of difficulty increased by altering the virtual mechanics of ball. Participants who completed 90 training trials reached the highest stage of task difficulty (level 30). The remaining two participants completed only 60 trials within the allotted time reached difficulty levels of 16 and 19 out of the maximum possible difficulty level. Mean CSE steadily decreased over all training trials (*r* = − 0.783, *p* < 0.001), which implies that with practice participants achieved the task using smaller lateral excursions (see Fig. 1E). In addition, movement time (MT) decreased with training (mean MT_1–10th trial_: 20.5 s; mean MT_final 10 trials_: 17.9 s; *r* = − 0.248, *p* = 0.019). Both movement parameters indicate that as participants advanced in training—they completed the trials faster and with fewer spatial errors.

### Effects on position sense acuity and movement accuracy in the trained wrist

**Figure 2:**
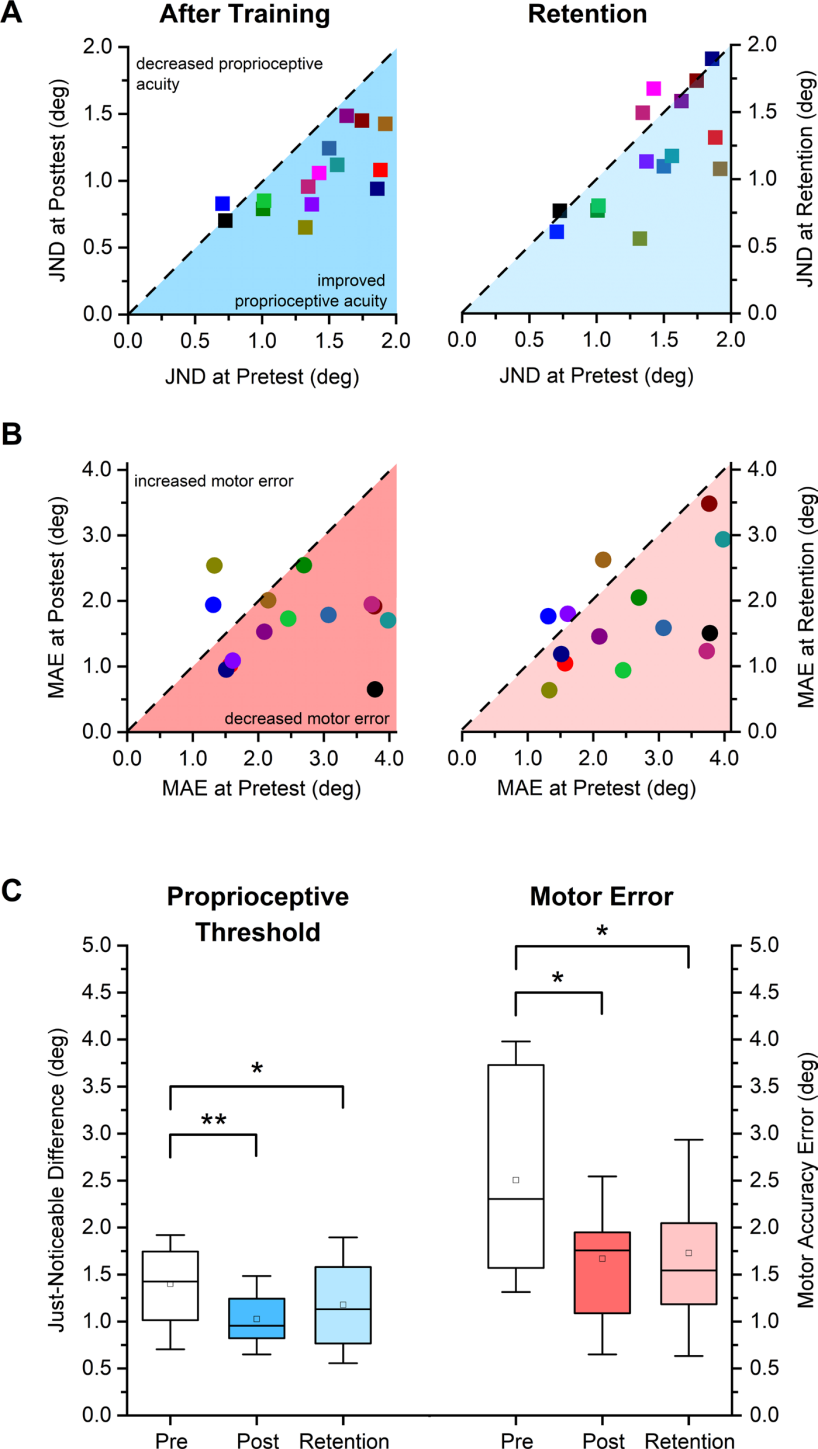
Sensorimotor effects of training on the trained right wrist as measured by the proprioceptive Just-Noticeable Difference threshold and the Movement Accuracy Error. Each colored symbol (square or circle) indicates the data of a particular participant after training and at retention. (**A)** JND thresholds for each participant at posttest and retention are mapped against their pretest thresholds. Dashed diagonal line represents the line of equality, i.e. indicating no change due to training. (**B)** Individual MAE values at posttest and retention are mapped against their pretest values. (**C)** Boxplot indicating the distribution of JND and MAE at pretest, posttest and retention. Boxes represent the range between 25 to 75th percentiles. Line within the box represents the median. The upper and lower whiskers extend to + 1.5 and − 1.5 inter-quartile range, respectively. **indicates *p* < 0.001, * indicates *p* < 0.05.

In order to understand the effects of the visuomotor training task on the trained right wrist, JND and MAE of all the participants at posttest were compared relative to pretest. The data in Fig. 2A illustrate that participants tended to exhibit a lower JND threshold after training (JND range at pretest: 0.71°–1.91°; at posttest: 0.70°–1.49°; see Fig. 2A). Mean JND decreased from 1.40° (SD: 0.39°) to 1.03° (SD: 0.27°). The corresponding mean relative change in JND between pretest and posttest was significant and yielded a large effect size (27% decrease, *t* = 5.11*, p* < 0.001, *d* = 1.32, see Fig. 2C). With respect to the performance in the untrained wrist-pointing task, 12 of the 14 participants (86%) showed gains in movement accuracy (see Fig. 2B; one outlier removed as value exceeded 2.3 times the interquartile range above the third quartile). Mean MAE was reduced from 2.50° (SD: 1.00°) to 1.67° (SD: 0.57°) at posttest, which corresponded to a significant mean relative change (33% decrease, *t* = 2.73* p* = 0.026, *d* = 0.73, see Fig. 2C).

To determine possible retention effects of training on the right wrist, JND and MAE of all participants at retention were compared. With respect to pretest, 11/15 participants (73%) retained the gains in proprioceptive acuity and 11/14 participants (79%) retained the gains in movement accuracy after 24 h (see Fig. 2A,B). A paired t-test between JND at posttest (mean: 1.03° ± 0.27°) and retention (mean: 1.18° ± 0.43°) revealed no significant differences in JND (*t* = − 1.68, *p* = 0.115, *d* = 0.43). The respective comparison for MAE at posttest (1.67° ± 0.57°) and retention (1.73° ± 0.80°) also failed to reach significance (*t* = − 0.251, *p* = 0.806, *d* = 0.07). Comparing the sensorimotor performance at retention relative to pretest, both JND (16% decrease, *t* = 2.70,* p* = 0.026, *d* = 0.70) and MAE (31% decrease, *t* = 3.17, *p* = 0.022, *d* = 0.85) were significantly reduced at retention (see Fig. 2C). In order to verify possible confounds due to exposure bias, we evaluated if MAE at the first three and last three trials at pretest were different. No such differences were found in both the wrists (right wrist: t = − 0.50, p = 0.62; left wrist: t = − 0.016, p = 0.88). In summary, these results indicate that training the right wrist enhanced its proprioceptive acuity and movement accuracy in the untrained motor task and these improvements were retained for up to 24 h.

### Proprioceptive and motor transfer effects in the untrained left wrist

**Figure 3:**
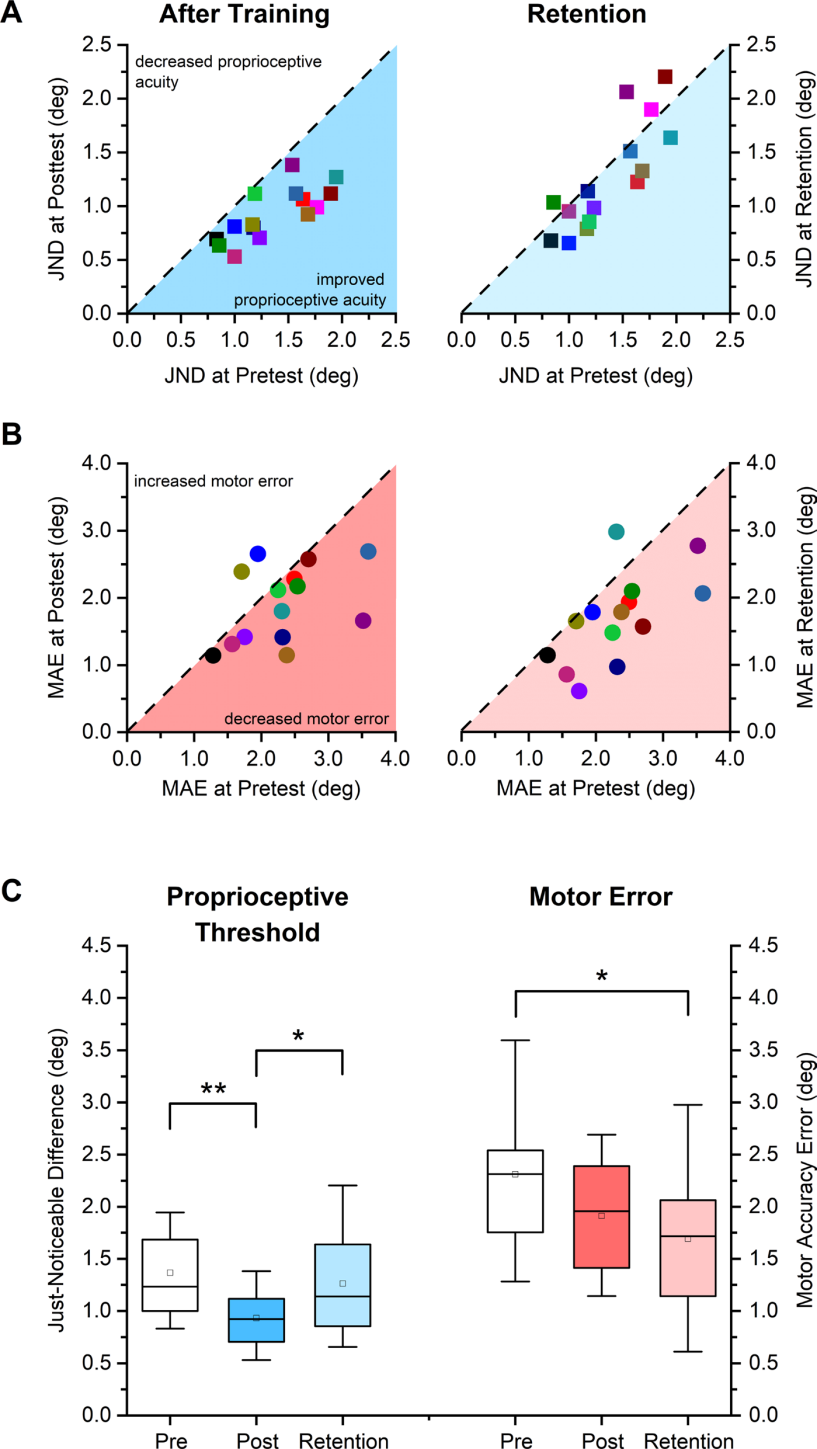
Sensorimotor effects of training on the contralateral untrained left wrist as measured by the proprioceptive Just-Noticeable Difference threshold and the Movement Accuracy Error. Each colored symbol (square or circle) indicates the data of a particular participant after training and at retention. (**A)** JND thresholds for each participant at posttest and retention are mapped against their pretest thresholds. Dashed diagonal line represents the line of equality, i.e. indicating no change due to training. (**B)** Individual MAE values at posttest and retention are mapped against their pretest values. (**C)** Boxplot indicating the distribution of JND and MAE at pretest, posttest and retention. Boxes represent the range between 25 to 75th percentiles. Line within the box represents the median. The upper and lower whiskers extend to + 1.5 and − 1.5 inter-quartile range, respectively. **indicates *p* < 0.001, * indicates *p* < 0.01.

In order to determine possible transfer effects of visuomotor training in the untrained left wrist, we analyzed JND and MAE for the left wrist at posttest. The within-subject change data between pre- and posttest of all participants are shown in Fig. 3A. The subsequent paired t-test and effect size analysis revealed that JND was significantly reduced from 1.37° (SD: 0.37°) to 0.93° (SD: 0.25°), which constituted a 32% decrease of JND at posttest (*t* = 6.86, *p* < 0.001) with a very high effect size (*d* = 1.77). The corresponding movement accuracy data for untrained the wrist-pointing task are shown in Fig. 3B. Mean MAE of the left wrist decreased by 17% from 2.31° (SD: 0.67°) to 1.91° (SD: 0.57°), yet this change was not statistically significant at group level (*t* = 2.20, *p* = 0.07; see Fig. 3C).

With respect to proprioceptive and motor performance at retention, the relevant within-subject pretest JND and MAE data are graphed against their retention values (see right panels in Fig. 3A,B). The respective paired t-test between posttest and retention revealed a significant rise in JND (36% increase, *t* = − 3.47, *p* = 0.006, *d* = 0.90) with mean JND rising to 1.26° (SD: 0.50°) at retention (from 0.93° at posttest; see Fig. 3C), indicating that the training-related gains in position sense acuity had significantly decayed 24 h after practice.

Motor performance of the wrist-pointing task at retention showed that mean MAE had increased to 1.69° (SD: 0.68°), but this increase was not significant with respect to posttest (*t* = 1.56, *p* = 0.269, *d* = 0.31; see right panel Fig. 3C). Comparing MAE means between pretest and retention revealed that this difference was significant (27% decrease, *t* = 4.00, *p* = 0.005, *d* = 1.07; see right panel Fig. 3C). In summary, these results indicate that position sense acuity and movement accuracy in the wrist-pointing task improved in the untrained left wrist as the result of right-wrist practice. At the 24-h retention assessment, gains in position sense acuity had significantly decayed, while gains in movement accuracy as measured by MAE had consolidated.

**Figure 4:**
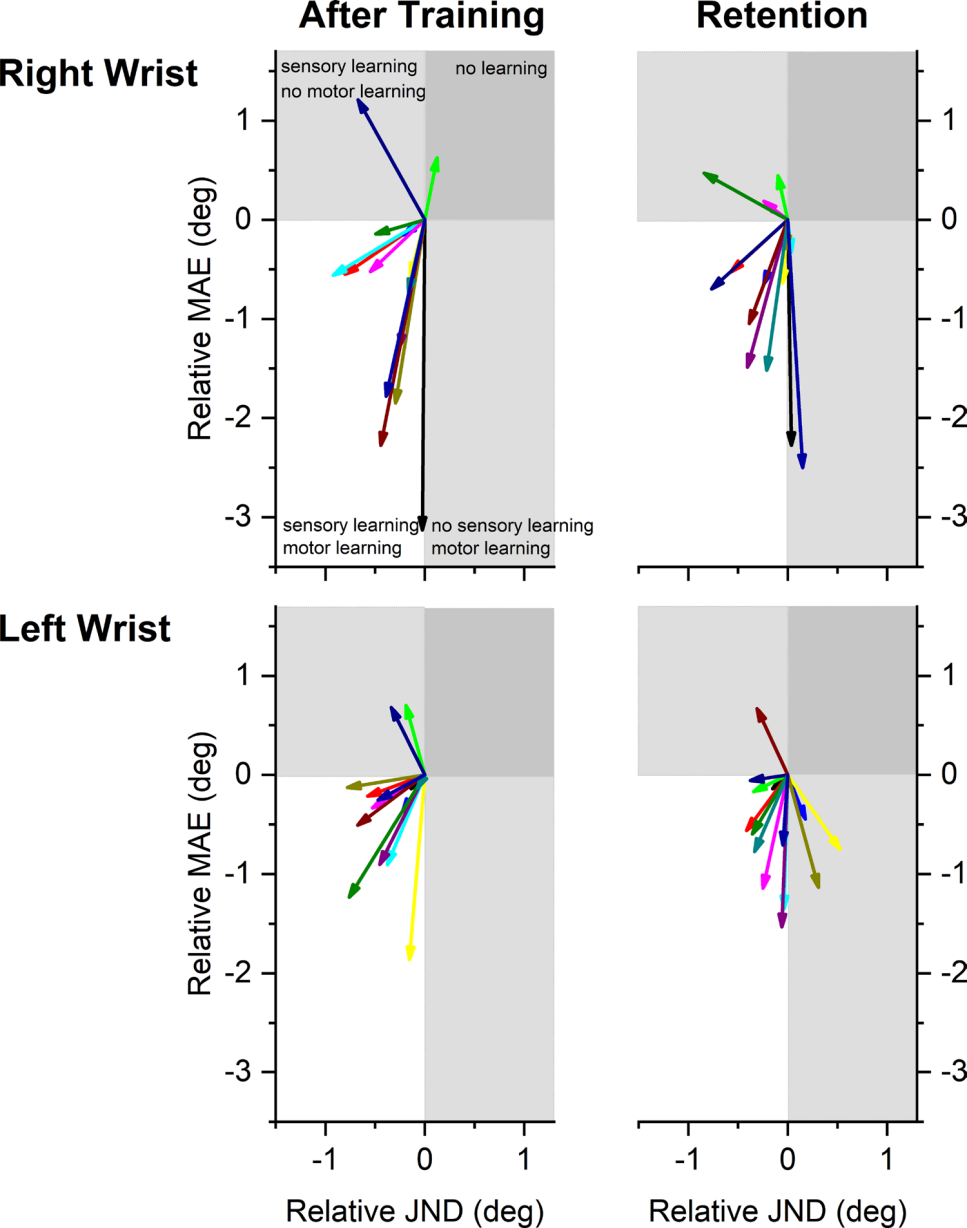
Vectorgram indicating the relationship between proprioceptive and motor learning at both wrist at posttest and retention. Each vector depicts a participant’s relative change in position sense threshold (JND) and spatial motor error (MAE) relative to pretest or retention. The coordinates of baseline JND and MAE are aligned to the origin (0, 0). Vector length indicates amount of relative change with respect to baseline. The lower left quadrant represents the zone where both proprioceptive and motor learning occurred.

To examine more closely the relationship between proprioceptive and motor learning for each participant, Fig. 4 maps individual changes in JND and MAE as a vector graph. Each participant’s learning gains with respect to JND and MAE at posttest and retention are represented as a vector. The origin (0, 0) represents the baseline or pretest performance level. The direction of the vector falls into one of four quadrants. A vector in the upper right indicate that both the proprioceptive threshold as well as the motor error increased at the end of training implying that no learning took place. Note that most vectors lay in the lower left quadrant indicating that proprioceptive and motor learning coincided and participants made concurrent gains in sensory as well as motor performance.

### Predictability of proprioceptive and motor transfer to the untrained wrist

It is important to understand whether the observed proprioceptive and motor function and its training-related changes in the right wrist can predict the observed changes in proprioceptive and motor function in the untrained left wrist. We therefore conducted multiple linear regression analyses to evaluate the relationship between the training-related changes in both the wrists. The change in JND at the right wrist (ΔJND) did not significantly predict ΔJND of the left wrist (R^2^ = 0.2; F = 3.278; *p* = 0.093). However, a model that accounted for the baseline levels in JND for both wrists and included ΔJND of the right wrist was and explained 65% of the variance seen in ΔJND of the left wrist (R^2^ = 0.654; F = 6.943; *p* = 0.007).

With respect to the change in motor error at the left wrist, a similar regression analysis revealed that ΔMAE left wrist was poorly predicted at 4% by ΔMAE of the right wrist (R^2^ = 0.047; F = 0.597; *p* = 0.455). The predictability of ΔMAE left wrist was best and significant with ΔMAE of the right wrist and the baseline MAE of both the wrists were entered as predictors into the model (R^2^ = 0.543; F = 3.963; *p* = 0.042).

## Discussion

The main purpose of the study was to investigate whether proprioceptive and motor learning at one limb system transfers to the contralateral homologous joint segment. The major findings of the study are as follows: First, there is initial evidence that learning-related gains in proprioceptive and untrained motor function at one limb system transfer to the contralateral limb. Second, the transfer of such learning occurs earlier in the sensory than in the motor domain. Gains in proprioceptive acuity were observed immediately after practice. In contrast, gains in motor performance of the untrained pointing task became prominent only at 24 h after training.

Previous work from our group documented that a proprioceptive-focused visuomotor training leads to enhanced proprioceptive acuity in the trained joint system, and importantly, such learning extends to parts of the motor domain that was not trained^5,6^. In other words, a proprioceptive-focused training of a motor task not only leads to improvements in the trained task, but learning can extend to other motor tasks executed by the same limb system. The neurophysiological basis behind such concurrent improvement in proprioception and untrained motor function are likely the reciprocal connections between the ipsilateral somatosensory and motor cortices. They mediate a bidirectional process of sensorimotor learning that is associated with concurrent experience-dependent neuroplastic changes in the motor and somatosensory system^8,10^ (for a review see^18^). For example, a recent neuroimaging study documented those learning-related changes in motor and as well as somatosensory cortex when humans learn a reaching movement to a hidden target. As they practiced and received positive reinforcement to promote learning, the functional strength of connectivity in a network linking motor (M1, PMd and SMA) and somatosensory cortices (S2) increased with more successful trials. Unsuccessful trials led to increased inter-trial movement direction corrections, which was associated with a reduction in connectivity in a network involving ipsilateral somatosensory cortex (S2), and contralateral supramarginal gyrus and lateral prefrontal cortex^19^. Sensorimotor learning is associated with changes in the motor and somatosensory system networks.

While the current study did not collect electrophysiological markers of sensorimotor learning, the psychophysical and kinematic data are consistent with previous research that reported gains in proprioceptive acuity (+ 34%) and motor accuracy (+ 27%) after a similar robot-aided proprioceptive training of the wrist^6^. These learning gains closely resemble previously documented reports, where both proprioceptive acuity (+ 27%) and motor accuracy (+ 33%) of the trained right wrist improved after training (see Fig. 2). The proprioceptive acuity typically observed in healthy adults is in the magnitude of about 10–15% of the reference position^20–22^. For instance, testing acuity at a 15° joint angle would result in discrimination thresholds of 1.5°–2.25°. In the current study, the training-related improvements reduced the proprioceptive acuity to about 6% of the reference position.

### Learning transfer to untrained left wrist

To our knowledge, this is the first report providing empirical evidence for an interlimb contralateral transfer of proprioceptive learning. Healthy human participants in our sample decreased their position sense discrimination thresholds of the untrained, left wrist/hand system by 32% at posttest. Given that we trained the right wrist/hand complex of people with a dominant right hand, it is remarkable that the size of the contralateral transfer of somatosensory measured at the left wrist is approximately of the same magnitude as the trained wrist (compare Fig. 2C with Fig. 3C).

When considering transfer of learning to another body hemisphere, it would be important to understand to what extent the gains of the trained limb predict the amount of transfer. Our data show that after accounting for the baseline performance of each wrist, the learning-related gains of the trained right wrist predicted about 65% of the change in proprioceptive acuity and 54% of the change in the motor error observed at the left wrist. This implies that higher learning gains at the trained limb are associated with a higher amount of transfer to the contralateral limb.

The neural mechanism underlying such transfer of proprioceptive and motor learning likely relies on interhemispheric connections via the corpus callosum. The majority of its fibers connect to homologous cortical regions in the contralateral brain hemisphere^23^ with the midbody of the corpus callosum mediating information transfer between the respective motor, somatosensory and auditory cortices^24^. Animal research has shown that besides callosal projections between homotopic regions of the primary somatosensory cortex (S1—areas 3b and 1)^25^, there are also callosal connections to the contralateral regions of the secondary somatosensory cortex (S2)^26^. The latter is important as current functional models propose that S2 is a major site for the integration somatosensory signals from both sides of the body (for a review see^27^).

While there has been no clear evidence that proprioceptive learning can transfer to the contralateral body hemisphere, there is research on tactile discrimination showing a somatotopically specific transfer with tactile perceptual learning. For example, when humans trained to discriminate pressure or roughness stimuli applied to a finger of the right hand (e.g. index finger), tactile acuity not only increased in the trained finger, but also in the adjacent fingers (e.g. right middle finger) and in the homologous finger of the left hand^28^. Interestingly, no signs of tactile learning were observed in the contralateral non-homologous fingers^29–31^. Given that touch perception and proprioception are both somatosensory modalities, the finding of a transfer of tactile learning to the contralateral, homologous body part is consistent with our finding that wrist proprioceptive acuity of the left wrist was enhanced after training the right wrist.

**Figure 5:**
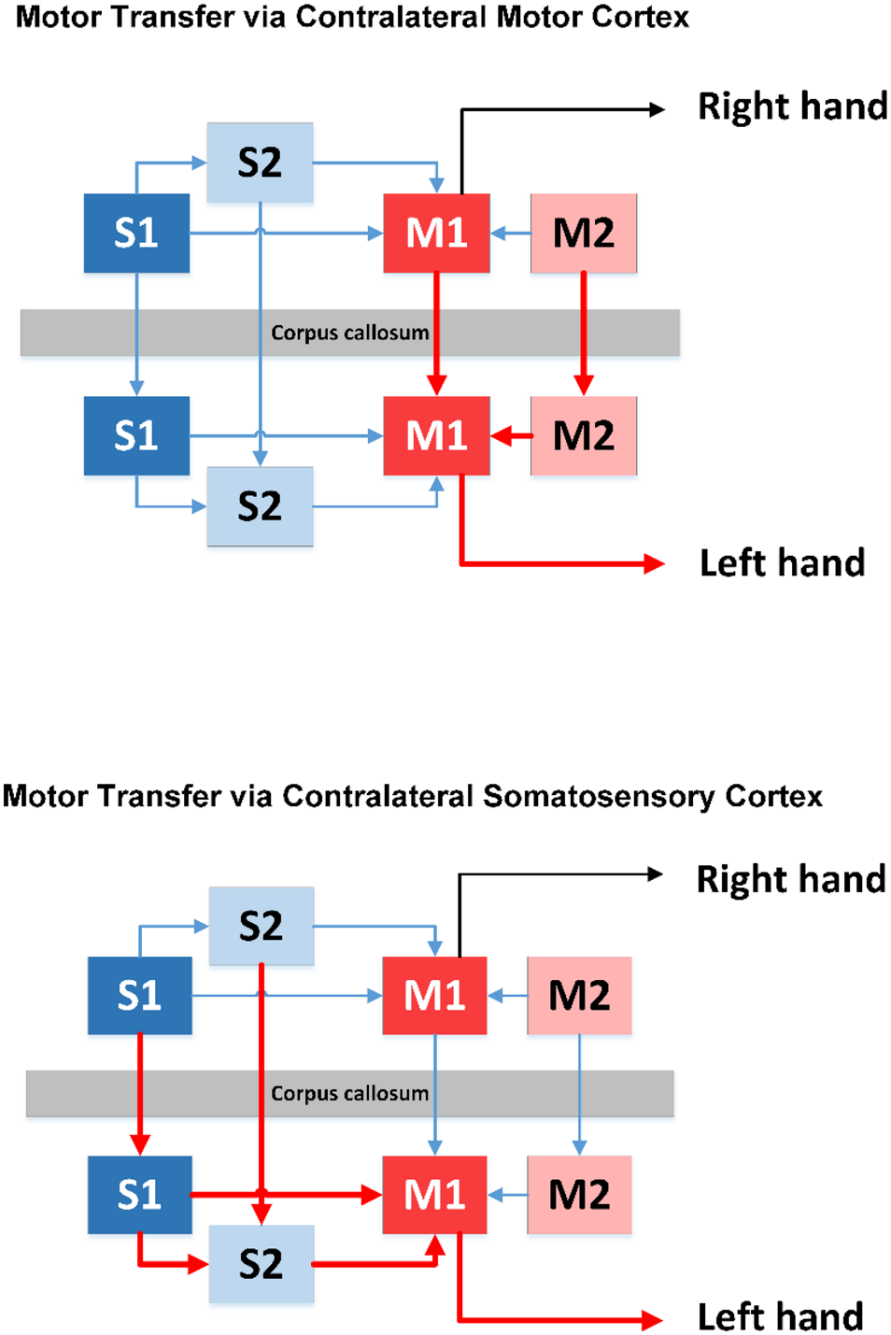
Possible neural pathways that explain the observed contralateral motor improvements. Top—Movement accuracy in the left hand can be observed through transcallosal projections between the homologous arm regions of left and right primary motor cortex controlling wrist motion. Bottom—Proprioceptive signals can be exchanged through transcallosal connections between the somatosensory cortices (S1 and S2) of each brain hemisphere and then transmitted to motor cortical neurons resulting in improved movement accuracy.

With respect to the observed contralateral motor effect, two scenarios of neural transmission can be envisioned. One pathway to account for the enhanced movement accuracy in the left hand is transmission via callosal projections between the homologous arm regions of left and right primary motor cortex controlling wrist motion. There is a body of research on bimanual coordination showing that interhemispheric interactions influence motor control and that motor training itself can modulate neural activity between the brain hemispheres^32^ (for a review see^33^). For example, learning a movement sequence task requiring key presses with the right hand did result in reduced interhemispheric cortical inhibition between M1 areas, which may contribute to a faster, more skilled performance of the contralateral hand^34^. An alternative pathway could relay proprioceptive signals via callosal connections between the somatosensory cortices (S1 and S2) of each brain hemisphere and then to motor cortical neurons (see Fig. 5). Regions of S1 (Brodmann areas 1, 2, 3a) are known to send strong efferent projections to the ipsilateral primary motor cortex, premotor cortex and the supplementary motor area^8^.

### Rate of learning and time course of memory consolidation

Our data show that significant changes in position sense acuity and movement accuracy in the wrist-pointing task were observable immediately after practice. This finding is consistent with earlier reports from our group showing evidence of fast proprioceptive and motor learning after only 30–40 min of practice^5^. What seems remarkable is that the transfer of proprioceptive learning to the contralateral wrist followed the same time course and improvement in the proprioceptive function was observed at the end of training. This implies that the short-term neuroplastic changes underlying such sensory learning must build up in a bilateral somatosensory cortical network during the learning process and that it does not take extra time after practice to consolidate sensory memory for the untrained, contralateral hemisphere. In contrast, motor learning followed a slower time course. The gains in movement accuracy were mild and not statistically significant at posttest (17% decrease in MAE), while they became significant 24 h later at retention (27% decrease in MAE) (see Fig. 3C).

Not surprisingly, a one-time training is not sufficient to induce lasting changes in sensorimotor learning. Within 24 h after practice, proprioceptive JND thresholds had regressed by 36% and the effect was no longer statistically significant at the group level. However, a previous report that employed a robot-aided wrist visuomotor training over 5 days documented that consolidation of proprioceptive learning was observed after 2 h at the end of each learning phase^4^. In addition, gains in somatosensory learning were retained over several days. Improvements in position sense acuity were retained for at least 3 days after training. This study further confirmed that somatosensory learning is closely linked to motor learning with both forms of learning showing memory consolidation and signs of cost savings over repeated sensorimotor training. That is to say, the learning gains made at one day do not disappear by the next day, but accumulate over repeated practice.

## Conclusion

This study documents that proprioceptive learning can transfer to the contralateral homologous limb segment. It complements previous work showing that tactile perceptual learning transfers to homologous body regions. In addition, untrained motor function improved slowly in the untrained left wrist, and showed significant improvement at 24 h after training. These findings provide a scientific basis for applying proprioceptive-based approaches to clinical populations such as stroke survivors. Given that people after a unilateral stroke experience proprioceptive and motor deficits in the more affected side of the body, a limb training with a focus of the less affected side could yield sensory and motor improvement in hemiplegic limb. However, at this point the learning-related gains for such population is unknown. Thus, before claiming the clinical significance of the approach systematic clinical trials are needed.

## Methods

### Participants

Fifteen healthy individuals (age: 24.67 ± 4.19 years; 8 males) with no known neurological conditions were recruited for study participation. In order to negate the potential confounding influence of handedness on proprioception and motor control, only right-handed individuals were included in the study. Handedness of the participants were determined by the Edinburgh Handedness Inventory^35^. The study protocol was reviewed and approved by the Institutional Review Board at the University of Minnesota’s Human Research Protection Program. The study was conducted in accordance with relevant guidelines and regulations. Written informed consent was obtained from all participants prior to data collection.

### Research design

The experiment followed a single-group, one-treatment design (see Table 1). Each participant completed pretest, posttest and retention assessments and a single training session in a span of three consecutive days. During each assessment session, wrist proprioceptive acuity and motor performance were evaluated bilaterally for each participant.

**Table 1:** Timeline and procedure of experimental protocol.

Day 1	Day 2			Day 3
**Pretest (40–60 min)** Proprioceptive & motor evaluation of right & left wrist	**Visuomotor training (30–45 min)** Right wrist only	**Break (20 min)**	**Posttest (40–60 min)** Proprioceptive & motor evaluation of right & left wrist	**Retention (40–60 min)** Proprioceptive & motor evaluation of right & left wrist

On day 1, participants completed the handedness questionnaire and the pretest assessment. Each participant completed the training session and the posttest assessment on day 2. On the third and final day, participants completed the same assessments as for pre- and posttest to determine retention of learning.

### Wrist robotic device

The wrist robot (see Fig. 1A) is a three degree-of-freedom exoskeleton allowing for the full range of motion (flexion/extension, adduction/abduction, and forearm supination/pronation) in the human wrist^20,36^. The wrist robot is a fully backdrivable system, powered by four brushless motors with the capability of delivering precise haptic, position and velocity stimuli at the wrist. The robot accurately encodes the wrist position at 200 Hz with a spatial resolution of 0.0075°. In addition, the robot is integrated with a virtual reality environment providing the user with a visual feedback of the user’s wrist position during the training session. The validity and reliability of this robot-based proprioceptive assessment have been established previously^20^.

### Procedure: visuomotor training

During the training session, participants sat comfortably with their right forearm resting on the support splint of the wrist robot and held the handgrip in a relaxed manner. Participants’ received visual feedback of their hand position in a monitor. Training required participants to balance a virtual ball rolling on a tiltable table within the virtual environment displayed in the monitor. Participants’ wrist position translated to the virtual table’s angle of inclination (see Fig. 1B,C). In each trial, participants’ goal was to balance the ball within a target zone by making precise, small amplitude, wrist flexion/extension movements. A trial was completed upon holding the ball within the target zone for 5 s^5,6^, after which a new target zone was presented. If the trial was completed within 60 s, it was considered successful. Between successful trials, the wrist position corresponding to the neutral, horizontal position of the table (where the ball would be stationary) was altered to either 10°, 15° or 20° of wrist flexion (where angular displacement is expressed relative to the neutral joint position). After a participant completed at least one successful trial in each of the three wrist flexion positions the task difficulty was automatically increased by altering the following virtual mechanical properties: (1) increasing the virtual mass of the ball and increasing the gain of the velocity of the virtual ball, and (2) decreasing the friction coefficients on the virtual table. Participants used a movement range of 10° wrist extension to 40° wrist flexion to complete the training trials (see Fig. 1D). To prevent fatigue, the training session was limited to a maximum of 90 training trials or 45 min. At optimal performance (every trial was successful) a participant would have completed 30 levels of difficulty. Participants were allowed a 2-min break after every 30 trials.

### Procedure: assessment and measurements

#### Evaluation of task-specific motor learning

In order to evaluate the effects of motor learning during the visuomotor task, all participants’ wrist angular time-series data were recorded using the signals of the position encoders of the robot. Instantaneous lateral deviation (LD) of the wrist from the neutral position was computed and cumulative spatial error for each trial was calculated using the equation below^6^:

1 $$CS{E}_{trial}={\int }_{i=1}^{n}\sum |L{D}_{i}|dt$$

where *n* is the last sample of each trial. Movement time was defined as the time of complete each trial. Cumulative spatial error (CSE) and movement time (MT) were derived for each trial. Changes in CSE and MT represented the measures to indicate performance changes during the training of the visuomotor task (i.e. task-specific motor learning; see Fig. 1B,C).

#### Evaluation of proprioceptive acuity

**Figure 6:**
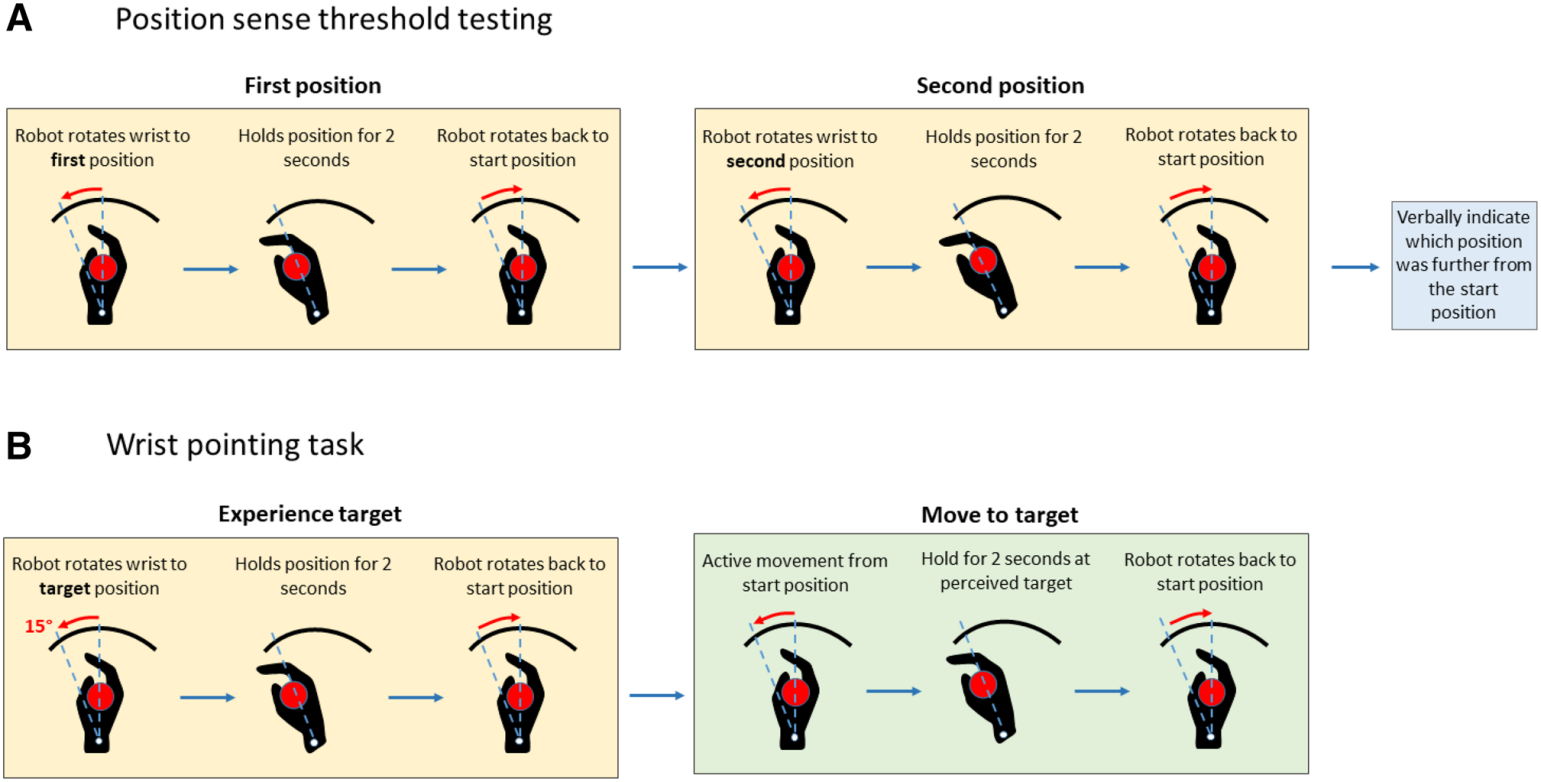
Proprioceptive function testing and evaluation of motor performance in the untrained motor task. (**A)** Schema of the procedure for position sense acuity evaluation. Shown is the testing of the right wrist. For testing, vision was occluded. No performance feedback was given between successive trials. (**B)** Schema of wrist-pointing task procedure to evaluate movement accuracy. Vision was occluded. For both wrist joints, participants actively flexed the wrist toward the perceived target position. No performance feedback was given between successive trials to avoid learning and solely test motor performance.

Proprioceptive acuity was evaluated in a wrist position sense discrimination task using the wrist robot. A psychophysical forced-choice paradigm was employed to evaluate wrist proprioceptive acuity. Participants wore opaque goggles and headphones playing white noise to block visual and auditory cues. In each trial, participants had to discriminate between two passively presented stimulus positions (a standard stimulus of 15° flexion and a comparison stimulus always greater than 15°, see Fig. 6A). The order of the standard and comparison stimuli presentation was randomized. The robot moved the wrist at a velocity of 6°/s from start (neutral position) to each stimulus position, held for 2 s, and then moved back to start. After both positions were presented, participants verbally indicated which position (first or second) was farther from the start position. The subsequent stimulus pair was then determined based on the verbal response by an adaptive psychophysical *psi-marginal* algorithm^37^. The complete evaluation consisted of 30 trials. Participants were allowed a 2 min break after 15 trials to avoid testing related fatigue. Participants’ verbal responses and the corresponding *stimulus difference size* (angular difference between comparison and stimulus positions) were recorded after every trial. Based on the verbal responses, a *Just-Noticeable Difference* (JND) threshold was determined by fitting the correct response rate and the stimulus difference size using a logistic Weibull function. The JND or the marginal threshold x slope posterior distribution was derived by summating across the lapse rate dimension using the following psychometric function:

$${p}_{\alpha ,\beta }\left(\alpha =a, \beta =b\right)=\sum_{l}p(\alpha =a, \beta =b,\lambda =l)$$

where p(α = a, β = b, λ = l) is the full posterior distribution defined across the threshold values a, slope values b, and lapse rate l values that are contained within the parameter matrix defined a priori. The resulting JND threshold represented a measure of proprioceptive acuity.

#### Evaluation of untrained motor performance

Motor performance was assessed by a discrete pointing task in the absence of vision. Vision and hearing were masked by use of opaque goggles and headphones playing a white noise. The wrist robot passively moved the participant’s wrist to 15° flexion from the start (neutral) position, held it for 2 s and then moved back to the start position. This allowed participants to experience the target based on proprioceptive information. Subsequently, participants actively moved the wrist to the perceived target position and held it for 2 s. Afterwards the wrist robot passively moved their wrist back to the start position (see Fig. 6B). The procedure was repeated for 10 trials. In each trial, the wrist robot recorded the angular position of the wrist joint during the 2-s hold period. Subsequently, the absolute angular error between the target position (15°) and the final joint position at the end of the pointing movement was computed for each trial. The mean absolute angular error across all trials for each participant was then calculated as *Movement Accuracy Error* (MAE) to represent a measure of untrained motor performance.

### Data analysis

For all participants, wrist position sense acuity and movement accuracy were evaluated bilaterally before training, and immediately and 24 h after training to determine the influence and the retention of right wrist visuomotor training. Data distributions for all variables were examined for normality using the Shapiro–Wilk test. Outliers were defined using the criteria of falling 1.5 times the interquartile range (IQR) below the first quartile or 1.5 times IQR above the third quartile^38^. Two outliers were identified in the MAE data, one for right and one for left wrist. These outliers were removed from further analysis. The remaining values for JND and MAE for both wrist data sets were normally distributed and parametric statistical analysis procedures were employed subsequently. Paired t-tests were performed on all comparisons for the right and left wrist to determine training related differences in JND and MAE between the three assessments. The initial significance level was set at *p* value = 0.05. To account for multiple testing, false discovery rate corrections using the Benjamini–Hochberg procedure were applied^39^. The correlation analysis focusing on how trained motor performance changed over the number of trials, we applied Spearman Correlation for CSE and Pearson-Product Correlation for MT. All statistical comparisons were performed by using the Statistical Package for Social Sciences (SPSS) version 24.0. Effect size (Cohen's *d*) and power calculations were computed using G*power 3.1.

## Data Availability

The datasets of the current study are available from the corresponding author upon reasonable request.
